# Recent Biotechnological Applications of Polyhydroxyalkanoates (PHA) in the Biomedical Sector—A Review

**DOI:** 10.3390/polym15224405

**Published:** 2023-11-14

**Authors:** Matheus Silva da Fonseca Diniz, Murilo Moraes Mourão, Luciana Pereira Xavier, Agenor Valadares Santos

**Affiliations:** Laboratory of Biotechnology of Enzymes and Biotransformations, Institute of Biological Sciences, Federal University of Pará, Belém 66075-110, Brazil; mouraomurilo@gmail.com (M.M.M.); lpxavier@ufpa.br (L.P.X.)

**Keywords:** polyhydroxyalkanoate, biodegradable, scaffolds, drug carrier, biomedical, plastics

## Abstract

Petroleum-derived plastics are materials of great importance for the contemporary lifestyle, and are widely used commercially because they are low cost, resistant, malleable, and weightless, in addition to their hydrophobic character. However, some factors that confer the qualities of these materials also cause problems, mainly environmental, associated with their use. The COVID-19 pandemic aggravated these impacts due to the high demand for personal protective equipment and the packaging sector. In this scenario, bioplastics are environmentally positive alternatives to these plastics due to their applicability in several areas ranging from packaging, to biomedicine, to agriculture. Polyhydroxyalkanoates (PHAs) are biodegradable biopolymers usually produced by microorganisms as an energy reserve. Their structural variability provides a wide range of applications, making them a viable option to replace polluting materials. PHAs can be applied in various biotechnology sectors, such as producing drug carriers and scaffolds for tissue engineering. This review aimed to survey works published in the last five years on the study and biotechnological application of PHAs in the biomedical sector, exploring the versatility and advantages of their use and helping to understand how to enhance their application.

## 1. Introduction

One of the most day-to-day materials used is plastic. This material is of great commercial importance since it has applications which range from industrial processes to the packaging of products. Plastic is of great economic importance due to its characteristics of being cheap compared to other materials, light, durable, and malleable, but several economic and social disadvantages are tied to using this material. The emission of greenhouse gases and environmental pollution are two of the problems associated with the use of plastic, mainly due to the linear flow in the value chain, as many applications are single-use products, such as packaging [1].

The versatility of plastics opens up the possibility of various applications, such as in straws, bottles, and plastic bags [2], and the packaging of food, pharmaceuticals, chemicals, and cleaning products [3]. The properties that make plastic an advantageous material are also some of the reasons why it is present in different ecosystems, making it a complex pollutant. The characteristic durability of plastics causes them to remain in the environment for hundreds or even thousands of years. Their hydrophobicity causes them to absorb contaminants, and their low weight allows the locomotion of plastics over long distances. Exposure to environmental factors, such as wave action and the sun, causes the breakdown of these plastics into microplastics (<5 mm), which facilitates their transport to even more environments, and microplastics are often consumed by organisms [4].

The importance of plastics on a day-to-day basis has boosted the increase in production to enable their supply across diverse applications. Thus, the production of 1.5 million tons recorded in the 1950s jumped to 359 million tons in 2018, with an increase in production between 63,000 and 430,000 tons in agricultural areas in Europe and 44,000 and 300,000 tons in North America [5]. From the total of this plastic, it is estimated that 50% of the total weight is composed of plastic materials intended for packaging, which is one of the most worrying residues of this material [6].

During the COVID-19 pandemic, the use of personal protective equipment (PPE), such as gloves and masks, increased considerably, in addition to the increase in other medical hospital waste. These increases overload the treatment facilities for SARS-CoV2 contaminated residues, causing the residues to have inappropriate destinations such as incineration, which is a potential source of greenhouse gases, in addition to other pollutants such as heavy metals. The increased consumption of single-use plastics for packaging and food packaging to avoid contamination was also significant. The increases from the period of social isolation tended to remain after confinement [7].

The pollution caused by these plastics makes strategies for better treatment to reduce the impact of these residues necessary. Circular economy strategies are one of the most common methods of changing the way plastics are disposed of through recycling. However, even this method presents problems, as recycled products can eventually become waste. In this context, chemistry and biotechnology present viable alternatives for treating residues through processes such as pyrolysis, biocatalysts, and the application of microbial cultures that, under appropriate conditions, are capable of degrading these plastics [8]. Another option to reduce the impacts is the replacement of petroleum-derived plastics with degradable biopolymers.

The substitution of these polluting materials contributes to the sustainable objectives established by the United Nations (UN), especially in objective number 12, linked to sustainable consumption and production, reducing the generation of waste, and encouraging companies to employ sustainable practices through the use of biodegradable materials in their production [9]. It also contributes to environmental, social, and governance (ESG) strategies adopted by companies to improve social responsibility, governance, and environmental best practices.

Biopolymers can be classified according to their origin, which includes natural sources, such as keratin, alginate, and collagen; microbial such as PHAs; and synthetic such as polylactic acid (PLA), polyethylene, and polystyrene. Furthermore, they can be classified as biodegradable or nonbiodegradable [10]. Among these polymers, PHAs have drawn attention due to their characteristics which provide products with great versatility, and for being biodegradable, conferring significant advantages over other biopolymers [11]. 

This review aims to explore the work carried out in the last five years related to the most diverse polyhydroxyalkanoates in the biomedical sector, to better understand the methodologies used and to emphasize the versatility of the biopolymer. A better understanding of how to use PHAs is desired to enhance economic viability and facilitate the optimization and cost reduction of the production process.

### 1.1. Polyhydroxyalkanoates

**Figure 1:**
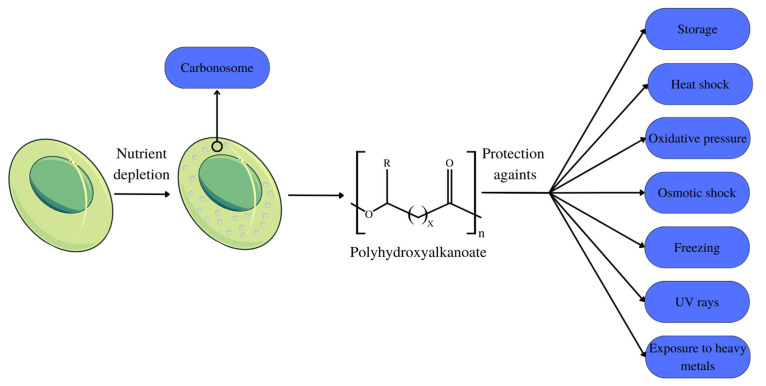
PHA production and cellular functions.

Polyhydroxyalkanoates (PHAs) are biodegradable biopolymers metabolically produced in nature in small quantities by various prokaryote organisms and some eukaryotes [12] as a form of storage component. They are stored in bacteria in the form of intracellular granules (Figure 1) [13]. Their diverse monomeric structure guarantees these polymers’ variable physical characteristics so that they serve different applications [14]. PHA is characterized into three groups according to the number of carbon atoms that make up their monomers: short-chain PHA, with 3–5 carbons in their structure; medium-chain PHA, with 6–14 carbons in their structure; and long-chain PHA, possessing a number of carbons greater than 14 [15].

PHAs are a class of polyesters characterized by ester bonds linking the carboxyl group of one monomer with the hydroxyl group of another. The majority of PHAs are produced in the R form [16]. Polyhydroxyalkanoate (PHA) molecules typically consist of a range of 600 to 35,000 monomer units. Approximately 150 distinct monomers have been recognized thus far, and this count is expected to increase as new types of PHAs are developed through chemical or physical modifications of natural PHA or via the utilization of genetically modified organisms [17]. The critical attributes exhibited by PHAs are the piezoelectric effect, nontoxicity, thermoplasticity, gas impermeability, hydrophobicity, enantiomeric purity, and a high degree of polymerization [18]. The multiple mechanical characteristics of polyhydroxyalkanoates (PHAs) arise from the range of monomers that may be included in their polymer structure, resulting in variations in flexibility, strength, and crystallinity. These properties exhibit variation based on factors such as the organism responsible for production, the number of carbons in a monomeric unit, and the specific growth circumstances [19].

Within microbial cells, PHA is present in the form of granules, also referred to as carbonosomes (Figure 1). These granules are complex molecules organized into layers, which are composed of different polypeptides, as well as proteins. Among the proteins present in the layers, phasins stand out, which can fill up to ¾ of the granule space, exercising a structural function by maintaining the spherical shape of the granules, in addition to participating in cell metabolism. The size of these granules varies between 100–500 nm, depending on the organism and the composition of the phasins that constitute the granule [20].

This versatility is essential in synthesizing and depolymerizing PHA molecules, promoting polymer accumulation, and determining granules’ size, quantity, and distribution. Other examples of phasins include PhaP1 from *Cupriavidus necator*, which can increase the activity of PhaC1 class II and PhaC2 from *Pseudomonas aeruginosa* in vitro, HAF from *Pseudomonas putida,* which regulates PHA synthesis, and ApdA from *Rhodospirillum rubrum* which plays a role in activating the depolymerization of Poly-(3-hydroxybutyrate) [20,21].

**Figure 2:**
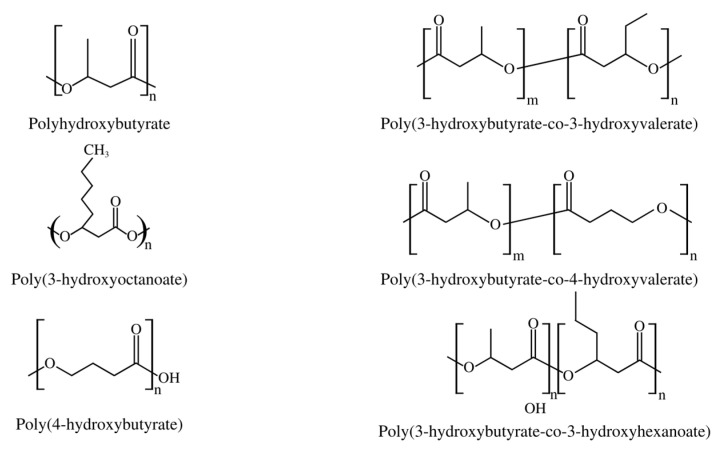
Examples of most common SCL-PHAs.

Short-chain PHAs (SCL-PHAs) exhibit high crystallinity, hardness, and fragility, but with slow crystallization rates and low impact resistance, along with rapid aging, making their use difficult. Poly-(3-hydroxybutyrate) (PHB), a homopolymer with four carbon subunits of 3-hydroxybutyrate (3HB), is the most frequent SCL-PHA, but other representatives of this group are poly(3-hydroxyoctanoate) (PHO), poly(4-hydroxybutyrate) (P4HB), poly(3-hydroxybutyrate-co-3-hydroxyvalerate) (PHBV), poly(3-hydroxybutyrate-co-4-hydroxybutyrate) (PHB-4HB), and poly(3-hydroxybutyrate-co-3-hydroxyhexanoate) copolymers (PHBHHx), as shown in Figure 2 [22].

Medium-chain-length PHA (MCL-PHA) polyhydroxyalkanoates exhibit distinct characteristics in comparison to SCL-PHA. This group is inherently elastomeric, more flexible than SCL-PHA, and has a lower melting point and is less crystalline. The primary producers of MCL-PHA are *Pseudomonas* sp., with β-oxidation as the production pathway in bacteria [20]. Additionally, the MCL-PHA has a lower glass transition temperature, low tensile strength, and excellent elongation until rupture [22]. 

PHAs are recurrently associated with their primary storage function. Nonetheless, a number of studies indicate that the functions of these polymers within the cell are more complex, including protection against stress factors such as thermal shock, oxidative pressure, osmotic shock, low temperatures, and freezing, as well as protection against the effects of UV rays and exposure to heavy metals [23]. During the growth phase of extremophile microorganisms, PHA accumulates to shield organelles from environmental stress and to support cellular functions [24].

### 1.2. PHA Synthesis

Some options for production improvement and the synthesis of new PHAs are the control of synthesis through the manipulation of regulatory elements such as enzymes and proteins and the engineering of biosynthesis pathways [25]. Consequently, it is necessary to comprehend these mechanisms in order to visualize the process in its entirety, as well as to use this knowledge to increase the yield and purity of the compounds produced.

**Figure 3:**
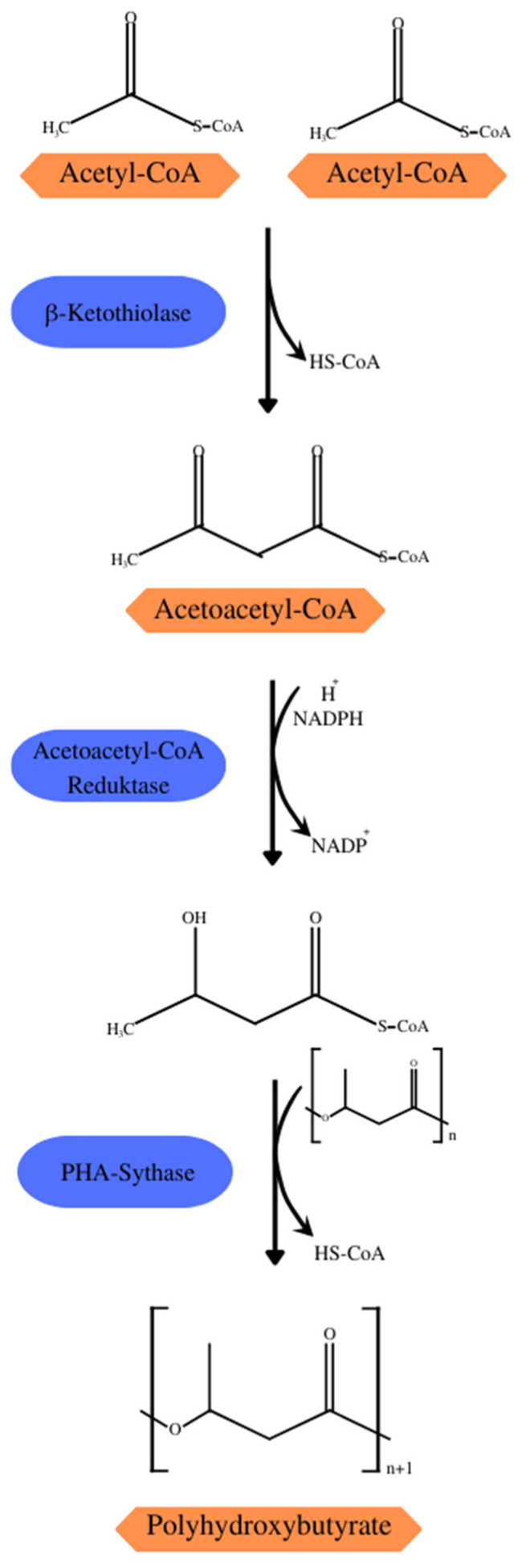
Synthesis of PHB with acetyl-CoA from the Krebs cycle as the precursor.

Some of the central metabolic pathways, such as the Krebs cycle, glycolytic and pentose–phosphate pathways, and degradation and biosynthesis pathways of fatty acids and amino acids, are directly or indirectly linked to the biosynthetic pathways of PHAs. The 14 pathways of PHA synthesis described so far demonstrate the importance of acetyl-CoA as a precursor to the biosynthesis of several SCL- and MCL-PHAs, such as PHB synthesis (Figure 3) which consists of three reactions catalyzed by acetyl-CoA acetyltransferase (β-ketothiolase; PHAA) catalyzing the condensation of two acetyl-CoA molecules into acetoacetyl-CoA, acetoacetyl-CoA reductase (PhaB) converting acetoacetyl-CoA into (*R*)-3-hydroxybutyl-CoA[(*R*)-3-HB-CoA], and PHA synthase (PhaC) that polymerizes (*R*)-3-HB-CoA in a PHB chain [21]. Under normal growth conditions, β-ketothiolase would be inhibited by the free coenzyme-A from the Krebs cycle. However, during the scarcity of noncarbonic nutrients in the substrate, the entry of acetyl-CoA becomes restricted, and the surplus is directed to the synthesis of PHB [26].

The synthesis of SCL-PHA occurs in Gram-positive, Gram-negative, and some haloarchaea species, with the majority of SCL-PHA producers able to accumulate PHB, and some species capable of producing the aforementioned copolymers. As stated earlier, acetyl-CoA plays a crucial part in the synthesis of SCL-PHA due to its function as a precursor for the 3HB monomer, which forms the PHB. Succinyl-CoA serves as the precursor for the synthesis of 4-hydroxybutyrate (4HB), an essential monomer. Conversely, 3-hydroxyvalerate (3HV) is derived from propionyl-CoA, acetyl-CoA, and, in certain instances, intermediates of β-oxidation of valerate or other fatty acids. The previously published mechanism for the production of 3HB monomer provides a broad understanding of the biosynthesis of SCL-PHA. Nevertheless, it is important to note that certain modifications may arise, particularly in situations when, in the first stage, condensation takes place between an acetyl-CoA and a propionyl-CoA molecule. This process results in the formation of 3-ketovaleryl-CoA subsequent to the reduction phase facilitated by 3-ketoacyl-CoA reductase, leading to the production of a molecule known as (R)-3-HV-CoA. The conversion of succinyl-CoA to succinate semialdehyde is facilitated by the activity of succinate semialdehyde dehydrogenase, which acts as a catalyst for this reaction. Subsequently, the molecule succinate semialdehyde undergoes oxidation through the action of 4-hydroxybutyrate dehydrogenase, leading to the formation of 4HB. In both cases, the monomers will ultimately be polymerized by the PHA synthases into PHB or one of its copolymers, PHBV, poly(3-hydroxybutyrate-co-4-hydroxybutyrate), and poly(3-hydroxybutyrate-co-3-hydroxyvalerate-co-4-hydroxybutyrate) (PHBV4HB) [25].

MCL-PHA can be synthesized in two ways: β-oxidation and new synthesis of fatty acids. These two pathways work interactively, with β-oxidation being the main route for the synthesis of MCL-PHA from fatty acids through their degradation, while the fatty acid synthesis pathway functions as the main metabolic pathway to obtain MCL-PHA from carbohydrates. The synthesis through related carbon sources (fatty acids) is more accessible than from unrelated carbon sources (carbohydrates), and the addition of fatty acids to the substrate with a structure similar to the monomer of the PHA can improve the synthesis of MCL-PHA [27].

The process of β-oxidation synthesis involves the enzymatic activity of (R)-specific-enoyl-CoA hydratase, acyl-CoA oxidase, and 3-ketoacyl-CoA reductase, which are then polymerized by PHA synthase to produce PHA [28]. One instance involves the operational mechanism of this specific pathway in *Aeromonas* spp., in which acyl-CoA derived from fatty acids undergoes degradation, resulting in the formation of enoyl-CoA intermediates. These intermediates can then be transformed into (R)-3-hydroxyoxycil-CoA through the activity of (R)-specific-enoyl-CoA hydrase. Subsequently, this (R)-3-hydroxyoxycil-CoA can participate in the elongation of the polyester chain by means of PHA synthase [29]. In the pathway of de novo fatty acid synthesis, an intermediate within the fatty acid synthesis pathway undergoes conversion by the transacylase PhaGPp into (R)-3-hydroxydecanoyl-CoA, which exists in its carrier protein form. This conversion process facilitates the utilization of (R)-3-hydroxydecanoyl-CoA as a substrate for the production of PHA [30]. The use of inexpensive substrates, such as glycerol residues, simple sugars, and short-chain volatile fatty acids derived from fermented residues, can be facilitated by this particular approach [31].

A fundamental enzyme in the synthesis pathways is PHA synthase or PhaC en-zymes, which is responsible for the polymerization of hydroxyacyl monomers to obtain PHA polymers. Several classes of PhaC with varying substrate specificities have been isolated from various microorganisms. PhaC of *Ralstonia eutrpha* (PhaCRe) is specific for SCL-PHA. The PHA synthases of *Pseudomonas* sp. are specific for MCL-PHA monomers. An additional example is the PHA synthase of *Aeromonas caviae*, which has a high affinity for units of 3HB and 3HHx and can therefore synthesize the copolymer P(3HB-co-3HHx) [32,33]. 

PHA is stored as an energy reserve when nutrients such as nitrogen, oxygen, and phosphorus are scarce or when the pH changes. The use of these energy stores for bacterial development occurs when the nutrition supply is restricted, as seen in Figure 1 [34]. This effect is demonstrated in mixed microbial cultures, with the limited nitrogen and phosphorus action resulting in the efficient production of PHA [35], just as the limitation of phosphorus and carbon was advantageous for the production of mcl-PHA [33]. The limitation of sodium nitrate and supplementation with sodium bicarbonate and sodium acetate optimized PHB production in *Stigeoclonium* sp. B23 microalgae [36].

## 2. PHA in Biotechnology

PHB is recognized for its applications in packaging and medical use (Table 1) due to its thermoplastic properties, but its fragility and high melting temperature limit its industrial use. In the present context, poly-4-hydroxybutyrate (P4HB) has demonstrated considerable potential as a polymer due to its inherent structural strength and high elongation capacity [20].

**Figure 4:**
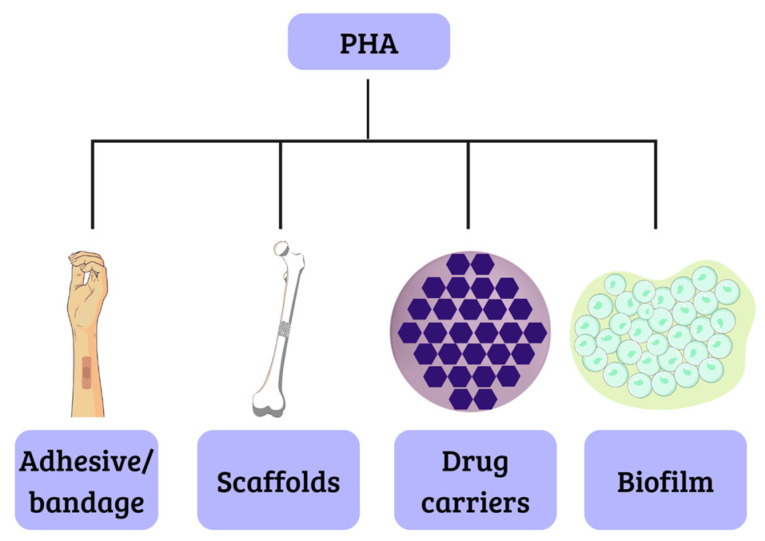
Biomedical applications of PHA.

This class of biopolymers generates considerable interest because they are biomaterials similar to latex or rubber, opening possibilities for applications in several areas including engineering, agriculture, food technology, pharmaceuticals, and, particularly, medicine and pharmacy. For instance, scaffolds have been manufactured with these materials successfully, due to their biocompatibility, which is achieved through the degradation of components that are naturally present in the body, like the polyhydroxybutyrate, that results in R-3-hydroxybutyric acid, a common constituent of blood. The biodegradability of these materials makes them suitable for the development of drug carriers. This is attributed to their low crystallinity and melting point, as well as their degradation through surface erosion. Additionally, they hold promise for applications such as electrocardiograph electrodes and adhesives for dressings [37,38], as illustrated in Figure 4.

The commercialization of PHA has been made since the 1980s; however, it has encountered several limitations and conditions that hinder commercial success. These difficulties are mainly associated with instability in thermomechanical properties and high production costs. The elevated costs can be attributed to the significant energy requirements caused by complex sterilization procedures and intensive aeration. Furthermore, the costly downstream processes, slow growth of microorganisms, and slow substrate (carbon) to product (PHA) rate of conversion contribute to overall high costs. Additionally, the discontinuous nature of these processes further adds to the cost of operation. In order to address these challenges, several studies have put forth various alternatives aimed at mitigating manufacturing expenses [39]. These alternatives include utilizing industrial residues as substrates for raw materials [40], chemically modifying PHA, employing genetic engineering techniques to enhance cell growth, manipulating cell morphology, and enhancing characteristics such as altering synthesis pathways [41].

**Figure 5:**
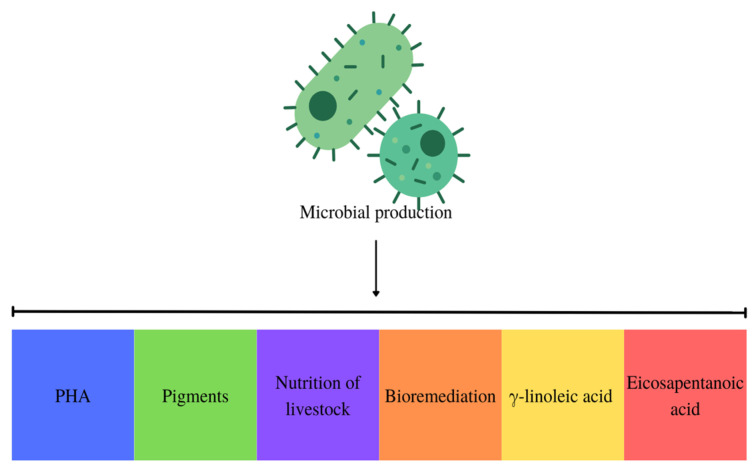
Obtaining PHA with multiproducts in the circular economy.

Implementing a circular economy throughout the microbial PHA manufacturing process represents a viable approach to mitigating expenses associated with bioplastic production. By integrating production with wastewater bioremediation, heavy metal and other waste removal, and environmental problem resolution, this approach enables the optimization of output while simultaneously achieving the desired product. An additional element in this approach that aids in the generation of PHA is the incorporation of this substance with other desirable products, such as pigments. Furthermore, residual biomass from cyanobacteria and microalgae used in animal feed production is utilized, and new microbial cultures are established, as illustrated in Figure 5 [42].

### 2.1. Scaffolds in Fabric Engineering

Tissue engineering offers solutions for repairing or replacing bones, nerves, cartilage, skin, heart valves, bladder, and blood vessels [43]. The multidisciplinary approach involving medicine, cell biology, and biomaterial engineering is part of the principle of tissue engineering. The scaffolds have a crucial role in providing three-dimensional structural support for cellular connections, facilitating the formation of the extracellular matrix (ECM), and serving as a carrier for growth factors and cytokines at the site of repair. The careful choice of a suitable biomaterial is of utmost importance in the development of a scaffold [44].

Scaffolds have the potential for treating bone defects by emulating the role of the extracellular matrix. They offer a three-dimensional environment that facilitates adhesion, proliferation, and differentiation, thus creating favorable physical circumstances for regeneration. Being biodegradable, biocompatible, osteoconductive, osteoinductive, and bioactive are conditions for the ideal scaffold [45].

The field of cardiovascular tissue engineering has made significant contributions to the management of heart diseases. It has played a crucial role in the development of transplant materials and the creation of biological models for preclinical pharmacological testing [46]. Additionally, it has proven to be essential in the structural repair of tissues with limited self-regeneration capabilities, such as tendons, ligaments, cartilage, and meniscus. Furthermore, tissue engineering applications may potentially involve corneas, parts of the urinary system, and bronchi as potential targets [47].

Polymers are one of the most crucial materials in the production of scaffolds due to properties such as hydrophobicity and biodegradability. PHA is used, both alone and combined, for biomedical applications of various types, such as sutures, patches, and dressings, as shown in the PHA produced by *Bacillus cereus* MCCB 281 [48,49].

The suitability of biopolymers P(3HB) and P(3HB-co-3HV) for the production of biocompatible fibrous scaffolds with a favorable shape for cell adherence was demonstrated using the electrospinning technique [50]. The viability of fibrous PHA scaffolds and films in promoting human cell proliferation has been demonstrated by Uribe, Acosta, and Díaz [51]. In addition, it has been demonstrated that these scaffolds and films do not induce toxicity in HEK 293 cells or embryonic renal cells. Furthermore, they have demonstrated resistance to autoclave and ultraviolet (UV) radiation, both of which are frequently utilized techniques for sterilization. The application of fibrous scaffolds has demonstrated improved effectiveness in stimulating cellular proliferation. After 18 days under cellular conditions, these scaffolds degraded, demonstrating their applicability in tissue engineering, where the scaffold degrades while the tissue regenerates.

The combination of PHA with other polymeric materials makes it possible to increase the efficiency of the physical–chemical properties of both polymers. The combination of PHA with additional polymeric substances enables the enhancement of the physical–chemical characteristics exhibited by both polymers. Bacterial cellulose (BC) is a applicable material for use in regenerative medicine due to its exceptional biocompatibility and biodegradability. When combined with PHB, BC demonstrated enhanced effects on osteoblast differentiation and induction of new bone formation, all while exhibiting high biocompatibility and no discernible signs of toxicity [52].

Another example of a combination of polymers is the immobilized PHB with gelatin (gel) and metronidazole (MTZ), a drug with antibacterial, antiprotozoal, and antiamebic capacity, which proved to be efficient in the production of scaffolds with the ability to increase the development of fibroblasts, while also inhibiting the growth of *Escherichia coli* and *Staphylococcus aureus*. The addition of the MTZ allowed increased compatibility with the L-929 fibroblast lineage, also providing good thermal and mechanical properties. The PHB/gel polymer in the proportion of 7:3 (m/m) added to 10% of MTZ showed the best results for skin regeneration, promoting the formation of myofibroblasts, regulating the inflammation of the wound and accelerating its regeneration [53].

The combination of PHB with gelatin and Fe_3_O_4_ is also viable and has been demonstrated as a feasible approach to produce magnetically-active hybrid scaffolds. These scaffolds have potential applications as dressings, in tissue engineering, and as drug carriers that can be regulated by an external magnetic field. Additionally, they are reported to be nontoxic to cells [54].

Scaffolds produced by the combination of poly(3-hydroxyoctanoate-co-3-hydroxyhexanoate) P(3HO-co-3HHX), an MCL-PHA, with hydroxyapatite, a compound with a structure similar to human bone used in bone implants, proved promising for bone tissue engineering, with excellent osteoconductivity and biocompatibility [55].

The combination of PHA with silver nanoparticles encrusted with graphene (GAg) demonstrated positive results for scaffolds intended to treat chronic injuries and sanitize applications due to their bactericidal potential [56]. Concurrently, positive results were observed with the growth and differentiation of neuronal tissues when PHA was combined with bioactive glass [57].

Three-dimensional printing technology is a viable alternative for producing PHA-based scaffolds, with positive results for potential drug carriers due to the incorporation of the osteogenic growth peptide, which accelerated cell differentiation [58].

Polymeric microspheres are a class of scaffold that offers ample area for cell-tying, enhanced by the 3D porous structure of these scaffolds, and can be kept in suspension. The interconnected porous structure also makes it easier to transport nutrients and metabolites, helping to proliferate and differentiate cells. Poly(3-hydroxylobutyrate-co-3-hydroxyhexanoate-co-3-hydroxyvalerate) (PHBVHHx) porous microspheres were made, and it was noted not only the characteristics mentioned but also a reduction in cell death and apoptosis due to their porous structure, making these microspheres promising for scaffolds and also as drug carriers [59]. The application of PHA in drug carriers will be further discussed next.

### 2.2. Drug Carriers

The first pharmaceutical–therapeutic application of PHA was probably in drug delivery systems (Figure 4). Studies have shown that the polymer is compatible with mouse fibroblasts, exhibiting no adverse effects on cellular growth and metabolism. Moreover, its ability to be recognized as a degradation product suggests its potential for natural elimination from the human body, hence positioning it as an advantageous option compared to other polymers [60,61]. Impallomeni et al. [62] and Mohandas et al. [49] showed promising results for the development of carriers from MCL-PHA produced by *Pseudomonas aeruginosa* ATCC 27853 and PHA copolymer produced by *Bacillus cereus* MCCB 281 using glycerol as a carbon source, respectively.

**Table 1:** PHA applications.

Polymer	Producing Body	Application	Author Year	Refs.
PHB	*Stigeoclonium* sp. B3	Drug carrier/biomaterial	Mourão et al., 2020	[36]
PHB	*Stigeoclonium* sp. B3	Drug carrier/biomaterial	Mourão et al., 2021	[40]
PHV	*Bacillus cereus* MCCB 281	Drug carrier	Mohandas et al., 2018	[49]
P(3HB)/P(3HB-co-3HV)/MCL-PHA	*Cupriavidus necator* DSM 428/*Pseudomonas chlororaphis* DSM 19603	Scaffolds	Esmail et al., 2021	[50]
PHB	*Azotobacter vinelandii*	Tissue engineering	Romo-Uribe et al., 2017	[51]
PHB	Unspecified	Scaffolds	Codreanu et al., 2020	[52]
PHB	Unspecified	Scaffolds	El-Shanshory et al., 2022	[53]
PHB	Unspecified	Scaffolds	Pryadko et al., 2022	[54]
P(3HO-co-3HHX)	Unspecified	Scaffolds	Ansari et al., 2017	[55]
PHA	Unspecified	Scaffolds	Lizzarraga-Valderrama et al., 2020	[57]
PHB	Unspecified (commercial)	Tissue engineering	Saska et al., 2018	[58]
PHBVHHx	Unspecified	Scaffolds	Wei et al., 2018	[59]
PHA	*Pseudomonas aeruginosa* ATCC 27853	Tissue/carrier engineering	Impallomeni et al., 2018	[62]
PHB	*Bacillus Cereus* VIT-SSR1	Drug carrier	Evangeline et al., 2019	[63]
PHB	Unspecified (commercial)	Drug carrier	Bini et al., 2017	[64]
PHB	*Pseudomonas aeruginosa* SU-1	Drug carrier	Senthilkumar et al., 2017	[65]
PHBV	Unspecified	Drug carrier	Vardhan et al., 2017	[66]
PHA	Unspecified	Drug carrier	Jiang et al., 2019	[67]
PHB	Unspecified	Drug carrier	Chen et al., 2021	[68]
PHA	Unspecified	Drug carrier	De Freitas E Castro et al., 2021	[69]
PHA	Unspecified	Drug carrier	Huerta-Angeles et al., 2017	[70]
PHA	*Bacillus subtilis* NCDC0671	Drug carrier	Umesh et al., 2018	[71]
P(3HO-co-3HD-co-3HDD)	*Pseudomonas mendocin* CH50	Drug carrier	Owji et al., 2021	[72]
poly(R-3-hydroxybutyrate-co-1,4-butylene adipate)	Unspecified	Drug carrier	Musumeci et al., 2019	[73]
PHBHHx	Unspecified	Drug carrier	Fan et al., 2018	[74]
P3HB	Unspecified	Drug carrier	Shershneva et al., 2018	[75]
PHA	Unspecified	Drug carrier	Canãdas et al., 2021	[76]
PHA	Unspecified	Drug carrier	Pavic et al., 2022	[77]
PHB	Commercial of bacterial origin	Biomedical use	Salama et al., 2018	[78]
P(3HV-co-3HB)	*Halomonas* sp.	Biomaterial	El-Malek et al., 2021	[79]
PHO	*Pseudomonas putida* KT2440	Wound treatment	Balcucho et al., 2023	[80]
P(3HB)/P(3HB-*co*-3HD)	*Pseudomonas mendocina* CH50 and *Bacillus subtilis* OK2	Wound treatment	Kalaoglu-Altan et al., 2021	[81]
PHA	*Pseudomonas chlororaphis* subsp. *aurantiaca*	Adhesive	Pereira et al., 2018	[82]
PHA	*Pseudomonas putida* KT2440	Adhesive and biofilm	Urbina et al., 2018	[83]
P(3HB)/P(3HB/3HV)	Unspecified	Herbicide carrier	Vijayamma et al., 2021	[84]

Mourão et al. [40] showed the potential for adaptive PHB production for drug carriers by the Amazonian microalgae *Stigeoclonium* sp. B23 using hydrolyzed cassava bark as substrate. The tests performed to characterize the PHB showed viability for biomedical application, having thermal, morphological, physicochemical, and biological characteristics similar to PHB and its copolymers produced by bacteria and cyanobacteria. The potential of this microalgae has been demonstrated by Mourão et al. [36].

The combination of PHB with chitosan resulted in a rigid matrix film, making drug carriers effective in releasing curcumin, a curcuminoid with anti-inflammatory, healing, and antioxidant properties widely used in the formulation of drugs [63]. Curcumin was also used in the work carried out by R.A. Bini et al. [64], where PHB nanoparticles were combined with a gelatin matrix to form a nanocomposite, being tested for the loading of curcumin, a hydrophobic drug, and naproxen sodium, a nonsteroidal and hydrophilic anti-inflammatory drug, with curcumin being carried by the PHB. In contrast, naproxen sodium was solubilized in the gelatinous matrix. This combination of the double release of drugs was favorable and has shown that a cheap, sustainable, and straightforward approach is practical for hydrophobic and hydrophilic drugs.

Drug release was reported using PHA produced by *Pseudomonas aeruginosa*, being loaded with curcumin in spherical nanoparticles with sizes between 300 and 500 nm, releasing the drug constantly for more than 5 h [65]. H. Vardhan et al. [66] demonstrated results that point to the nanocomposite composed of PHBV as an alternative to improve breast cancer treatment through the loading of the hydrophobic drug docetaxel. The drug-pumping system applied to fight cancer using docetaxel has also been shown to be effective using a thermogel based on poly[(R)-3-hydroxybutyrate-(R)-3-hydroxyhexanoate] copolymerized with poly(ethylene glycol) and polypropylene glycol. The application of this drug, combined with the carrier, proved to be effective in treating melanomas without causing damage to healthy tissues, proving to be a promising carrier for anticancer drugs [67].

The incorporation of PHA in conjunction with other polymers offers a promising alternative to drug-pumping systems. The junction between PHA and polyvinyl alcohol (PVA) is an example where the specificities of both polymers complement each other, reducing the degradation rate of PVA. Additionally, the PHA membrane supports water-soluble PVA fibers. The efficacy of this combination was demonstrated in the transportation of levofloxacin, resulting in highly efficient bactericidal results against Gram-negative strains of *E. coli* and Gram-positive strains of *S. aureus* [68]. Furthermore, the incorporation of compounds can also serve to modulate the release of the drug by the carrier, as well as the incorporation of superparamagnetic nanoparticles. A carrier with a thermal responsive property regulated by magnetic oscillation was demonstrated to be a promising candidate for veterinary application when PHA copolymers were combined with nanomagnetite to achieve controlled progesterone release [69]. G.H. Angeles et al. [70] also described an effective combination in encapsulating hydrophobic drugs, which is the copolymer formed by combining hyaluronic acid and PHA. 

*Bacillus subtilis* was able to produce PHA from a culture medium containing orange peel as a way to reduce the cost of polymer production. This PHA produced was proved effective in administering levofloxacin, with the release of the drug reaching up to 99.12% [71]. The bacterium *Pseudomonas mendocina* CH50 showed positive results for the production of poly(3-hydroxyoctanoate-co-3-hydroxydecananoate-co-3-hydroxydodecanoate) or P(3HO-co-3HD-co-3HDD). This polymer was used as a film and submitted to a coating process based on the chemical used by mussels to adhere to wet surfaces, thus allowing it to overcome the main barrier for the targeted application of drugs in the oral mucosa. The polydopamine lining showed positive results, increasing film adhesion in in vitro cell proliferation and in in vivo neovascularization [72]. T. Musumeci et al. [73] showed that poly(3-hydroxybutirato-co-butylene adipate) can produce drug nanocarriers. 

The formation of PHA nanoparticles can also be performed using the interaction of the polymer with the phasins. PHBHHx was combined with PhaP phasin modified with a targeting peptide that recognizes an epidermal growth factor receptor to develop a targeting system for tumors, proving to be suitable as a drug carrier [74]. The spray-drying technique was used with efficiency in the production of drug carriers. Microparticles of P3HB and P3HB were combined with polyethylene glycol(PEG) for the paclitaxel(PTX) and 5-Fluorouacil(5-FU) drugs carriage. The method showed improvement in yield and greater incorporation of drugs into microparticles, and extended release of drugs led to inhibition of tumor cell growth [75]. PHA was also utilized as an inhaled drug carrier for the lungs; however, additional research is required to determine the viability of these carriers [76].

As drug carriers, the PHA microspheres that were previously mentioned as scaffolds also demonstrated promising results. A. Pavic et al. [77] showed the feasibility of using MCL-PHA in microspheres to transport antifungal drugs to combat candidiasis. These microspheres loaded with polyene demonstrated excellent antifungal activity and decreased the toxic effects of these drugs on zebrafish embryos, being a viable alternative to combat candidiasis; however, further investigation is required. 

### 2.3. Other Applications in Medicine

Polyhydroxyalkanoates have other biomedical applications beyond the production of scaffolds and drug carriers. Salama, Aziz, and Saad [78] demonstrated a process of incorporation of silver nanoparticles into a PHB and chitosan copolymer. Silver nanoparticles’ antimicrobial activity led to positive results regarding the Gram-negative bacteria *Escherichia coli* and *Salmonella typhi*, Gram-positive *Streptococcus pneumoniae*, and the fungus *Aspergillus fumigatus*. In addition, chitosan is essential for this outcome, which opens possibilities for the use of this compound in various sectors of the biomedical area. Poly-based nanoparticles (3 HV-co-3HB), produced by *Halomonas pacifica* ASL10 and *Halomonas salifodiane* ASL11 using substrates derived from algae hydrolysate, presented antibacterial characteristics that make them suitable for use in the pharmaceutical industry and food packaging [79].

Another application for PHAs is the treatment of wounds and injuries. Some works report the efficiency of the PHAs, especially blended with other materials. Poly(3-hydroxyoctanoate) showed positive results when incorporated with Ag nanoparticles for antimicrobial wound dressing on the treatment of skin and soft tissue infections, being effective against methicillin-resistant *Staphylococcus aureus*, in addition to nontoxic biodegradation, reducing environmental impact and being suitable for long-term applications [80].

An additional function of PHAs is the treatment is the blend of the SCL-PHA P(3HB) and the MCL-PHA P(3HB-*co*-3HD), combining the properties of both polymers, making it suitable for the confection of wound dressing. These nanofibers were incorporated with AgNPs via a dip-coating method, contributing to cellular metabolism in the wound-healing process [81].

J.R. Pereira et al. [82] describe the production of MCL-PHA from crude glycerol obtained from biodiesel production. The resulting polymer exhibits reduced hydrophobicity and crystallinity, which makes it suitable for the production of elastic and flexible films (Figure 4). Consequently, this material finds potential applications in the biomedical field, particularly in the development of injury treatment materials. This aligns with the findings of Urbina et al. [83], who also investigated MCL-PHA properties. Furthermore, this material demonstrated use in the biomedical sector and the manufacturing of adhesives (Figure 4). Additionally, its elastic, adhesive, and soft properties at room temperature made it suitable for implementation in the field of agriculture.

An application similar to drug delivery was reported by R. Vijayamma et al. [84], where a herbicide delivery system composed of P(3HB) and P(3HB/3HV) was tested. Metribuzin, a herbicide derived from 1,2,4-triazine, demonstrated the best release results with an index of 44–48%, and in the tests carried out during the growth of the plant, *Elsholtzia ciliata* microparticles loaded with metribuzin and tribenuron-methyl demonstrated the best result. 

## 3. Conclusions

Polyhydroxyalkanoates are highly versatile, having applications in several areas, such as in the biomedical sector, and are widely used in scaffolds in tissue engineering, enabling aid in the regeneration of various tissues and adding antimicrobial and anti-inflammatory properties when combined with other materials. Their use in drug carriers is also one of the most common applications due to biocompatibility, due to their degradation resulting in products natural to the body, and biodegradability, which enables the direct application of drugs in the desired tissues, increasing the applicability of the compound and reducing the inherent toxicity of some drugs, and being more attractive than other polymers due to their capacity to be recognized by the human body as degradation product, which is naturally removed from the body. Thus, these polyesters are shown as a viable option for replacing petroleum-derived plastics, reducing the environmental impact caused by them. The economic barrier caused by the high production costs of these polymers is still an obstacle to be faced, as shown in several studies, thus being necessary for further studies to reduce the cost of production and become more commercially attractive by methods like biorefinery, which make it possible to obtain multiple products combined in PHA production, associating the production with bioremediation of residual waters and improving the use of microorganisms that require lower costs in their cultivations, such as microalgae that can be cultivated in residual water and sludge and require fewer nutrients due to their autotrophic behavior.

## Data Availability

The data presented in this study are available on request from the corresponding author.
